# Association of Hormone Receptor Expression with Survival in Ovarian Endometrioid Carcinoma: Biological Validation and Clinical Implications

**DOI:** 10.3390/ijms18030515

**Published:** 2017-02-27

**Authors:** Peter Rambau, Linda E. Kelemen, Helen Steed, May Lynn Quan, Prafull Ghatage, Martin Köbel

**Affiliations:** 1 Department of Pathology and Laboratory Medicine, University of Calgary, Calgary, AB T2N 2T9, Canada; peter.rambau@ucalgary.ca; 2 Department of Pathology, Catholic University of Health and Allied Sciences, P.O. Box 1464, Mwanza, Tanzania; 3 Department of Public Health Sciences, College of Medicine, Medical University of South Carolina, Charleston, SC 29425, USA; lkelemen@post.harvard.edu; 4 Hollings Cancer Center, Medical University of South Carolina, Charleston, SC 29425, USA; 5 Department of Obstetrics and Gynecology, University of Alberta, Edmonton, AB T5H 3V9, Canada; Helen.Steed@albertahealthservices.ca; 6 Division of General Surgery and Surgical Oncology, University of Calgary, Calgary, AB T2N 2T9, Canada; MayLynn.Quan@albertahealthservices.ca; 7 Department of Gynecological Oncology, Tom Baker Cancer Centre, University of Calgary, Calgary, AB T2N 2T9, Canada; Prafull.Ghatage@albertahealthservices.ca

**Keywords:** ovarian cancer, endometrioid, estrogen receptor, progesterone receptor, hormonal therapy, prognosis

## Abstract

This paper aims to validate whether hormone receptor expression is associated with longer survival among women diagnosed with ovarian endometrioid carcinoma (EC), and whether it identifies patients with stage IC/II tumors with excellent outcome that could be spared from toxic chemotherapy. Expression of estrogen receptor (ER) and progesterone receptor (PR) was assessed on 182 EC samples represented on tissue microarrays using the Alberta Ovarian Tumor Type (AOVT) cohort. Statistical analyses were performed to test for associations with ovarian cancer specific survival. ER or PR expression was present in 87.3% and 86.7% of cases, respectively, with co-expression present in 83.0%. Expression of each of the hormonal receptors was significantly higher in low-grade tumors and tumors with squamous differentiation. Expression of ER (Hazard Ratio (HR) = 0.18, 95% confidence interval 0.08–0.42, *p* = 0.0002) and of PR (HR = 0.22, 95% confidence interval 0.10–0.53, *p* = 0.0011) were significantly associated with longer ovarian cancer specific survival adjusted for age, grade, treatment center, stage, and residual disease. However, the five-year ovarian cancer specific survival among women with ER positive stage IC/II EC was 89.0% (standard error 3.3%) and for PR positive tumors 89.9% (standard error 3.2%), robustly below the 95% threshold where adjuvant therapy could be avoided. We validated the association of hormone receptor expression with ovarian cancer specific survival independent of standard predictors in an independent sample set of EC. The high ER/PR co-expression frequency and the survival difference support further testing of the efficacy of hormonal therapy in hormone receptor-positive ovarian EC. The clinical utility to identify a group of women diagnosed with EC at stage IC/II that could be spared from adjuvant therapy is limited.

## 1. Introduction

Approximately 80% of ovarian endometrioid carcinomas (ECs) present with disease confined to the pelvis (stage I and II) [1]. EC carries the most favorable prognosis among the ovarian carcinoma histotypes with a five-year survival rate of more than 70% across all stages. For patients diagnosed at sub-stage IA/IB or FIGO (International Federation of Obstetricians and Gynecologists) 2014 IC1 (IC with surgical spill only) the five-year survival is about 95% and those patients do not require adjuvant therapy after surgery [2,3]. In order to identify more patients that can safely be spared from toxic chemotherapy, molecular prognostic markers may be of aid.

The molecular composition of EC has been recently explored. EC harbors mutations in *CTNNB1*, *PIK3CA*, *KRAS*, *ARID1A*, *PTEN*, *PPR2R1A* but uncommonly in *TP53* (less than 15%) [4]. Mismatch repair protein expression is deficient in 10%–13% of cases [5,6]. WT1 expression is typically absent in EC (although expressed in up to 10% of cases). Despite an excessive amount of literature on prognostic markers in ovarian carcinoma in general, none are in clinical use. One issue, which relates to EC in particular, is the shift in criteria for this histotype diagnosis over the last decade. Today, many of the formerly diagnosed “high-grade endometrioid” carcinomas are classified as high-grade serous carcinoma, which is supported by molecular evidence (e.g., combination of WT1 expression with *TP53* mutation or *BRCA1/2* mutations) [7,8,9,10]. As a consequence, contemporarily classified retrospective EC cohorts are sparse. 

Hormone receptors are frequently expressed in ovarian carcinomas, and a recent meta-analysis of 35 studies reported that progesterone receptor (PR) expression is associated with longer survival [11]. Most of these studies included all ovarian carcinoma histotypes. However, due to the association of certain markers with certain histotypes, outcome associations are confounded (e.g., high-grade serous carcinomas frequently express estrogen receptor (ER) but are associated with an unfavorable outcome) [12]. We recently showed in a histotype-specific analysis using a large pooled cohort from the international Ovarian Tumor Tissue Analysis (OTTA) consortium that ER and PR expression was associated with improved survival specifically in EC (*n* = 484) [13]. However, in this study histotype assignment was not standardized. 

We hypothesized that ER or PR expression is associated with improved outcome independent of main risk factors using an independent cohort, which was rigorously classified for histotype. The secondary hypothesis was whether ER/PR expression status might inform prognostication in a stratified analysis by sub-stage. Further, we explored whether ER/PR expression status might predict benefit from adjuvant therapy.

## 2. Results

### 2.1. Study Cohort and Patient Characteristics

We identified 207 endometrioid carcinomas diagnosed in Alberta, Canada between 1979 and 2010 from the Alberta cancer registry [14]. For 182 cases diagnosed between 1989 and 2010 tissue was available and EC histotype was confirmed based on a retrospective two-step procedure. First, two gynecological pathologists reviewed the full H&E slides sets. Second, an 8-marker immunohistochemical algorithm for ovarian carcinoma histotype/subtype probability was applied. The details of reclassification have been previously described [10]. Patients with confirmed EC were on average 54 years old ranging from 25 to 91, and 29.7% of women were pre-menopausal. The mean body mass index (BMI) was 29.4 kg/m^2^ (SD ± 7.7), and 40.0% of the patients were obese (BMI ≥ 30 kg/m^2^). Diagnosis and treatment were almost equally distributed between the two main cancer centers in Alberta. At diagnosis, 32.0% of patients had ascites. The mean CA125 level before treatment was 535 kU/L (SD ± 987) and 85.2% were considered abnormal using a cut-off of ≥35 kU/L. Seven patients received neoadjuvant chemotherapy. Salpingo-oophorectomy was performed in all patients, which was bilateral in 87.4% and unilateral in the remainder. The ovarian tumors were on average 11.3 cm in size (SD ± 5.9). Variable staging procedures included pelvic lymph node sampling (81.9%), para-aortic lymph node sampling (34.1%), omentectomy (86.8%), appendectomy (12.6%), and segmental colon resection in (3.3%). Tumor rupture during surgery occurred in 32.4% of the patients and 4.9% of the patient had a ruptured tumor before surgery. Macroscopic residual tumor was present in 8.2% after initial surgery. The residual tumor status was not documented in 40.1% of patients. On pathology review, less than 20% of EC were grade 3. Three cases showed features of dedifferentiation. Roughly half of ECs showed evidence of squamous differentiation. Most patients were diagnosed at stage I (51.7%) or stage II (35.0%). Endometrioisis was present in 58.2% of patients.

First line adjuvant chemotherapy was given to 115 (63.5%) of patients. The first line chemotherapy drugs were carboplatin (78%), cisplatin (19%), and melphalan (3%). Paclitaxel was given in combination to 71% of patients receiving chemotherapy. Radiation was administered to 18 patients (10%), 6 received radiation alone, and 12 in combination with chemotherapy. The mean follow-up time for censored patients was 112 months (SD ± 49.3). In 45 (25%) of patients, the disease recurred and 51 patients died during the follow up time. Five patients died of treatment related causes (3%) and eight patients due to other causes unrelated to ovarian cancer. Thirty-eight patients (21%) died due to ovarian cancer. The five-year ovarian cancer specific survival (OCSS) in relation to selected clinical variables is shown in Table 1.

### 2.2. Hormonal Receptor Expression

A single case had insufficient tumor tissue present on the tissue microarray resulting in 181 evaluable cases. Twenty-three (12.7%) cases were negative for ER, 26 (14.3%) showed focal staining, and 132 (73.0%) showed diffuse staining. By combining the latter two, ER expression was present in 158 (87.3%) of ECs. Twenty-four (13.3%) cases were negative for PR, 23 (12.7%) showed focal staining, and 134 (74.0%) showed diffuse staining. By combining the latter two, PR expression was present in 157 (86.7%) of ECs. Co-expression of ER and PR was detected in 151 (83.4%) cases; 7 (4.4%) were ER+/PR−, 6 (3.3%) were ER−/PR+, and 17 (9.4%) were ER−/PR−. ER and PR expression was more commonly seen in grade 1 EC, tumors with squamous differentiation, and associated with a higher BMI, but no other features (Table 2).

### 2.3. Hormonal Receptor Expression and Association with Survival

**Figure 1:**
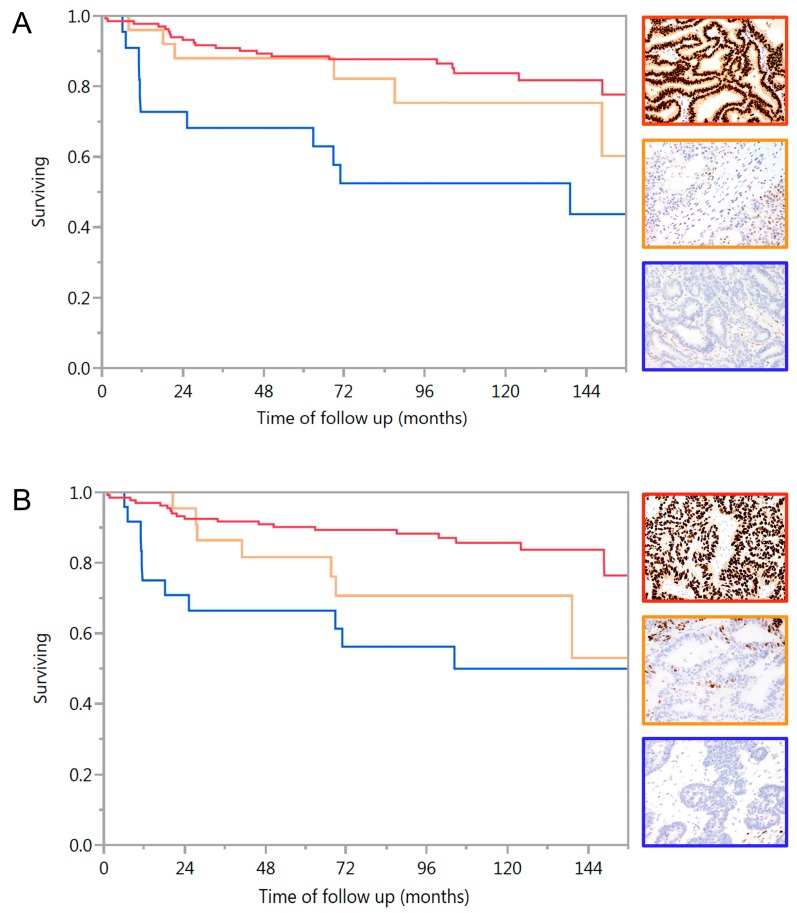
Kaplan-Meier curves of ovarian cancer specific survival for endometrioid carcinoma by hormone receptor status. (**A**) Survival by estrogen receptor (ER) (log rank *p* = 0.0005) red line and red framed image: diffuse ER expression; orange line and orange framed image: focal ER expression, blue line and blue framed image: absence of ER expression in tumor epithelium (note positive intrinsic control); (**B**) Survival by progesterone receptor (PR) (log rank *p* = 0.0003) red line and red framed image: diffuse PR expression (note absence of PR expression in areas of squamous differentiation); orange line and orange framed image: focal PR expression, blue line and blue framed image: absence of PR expression in tumor epithelium (note positive intrinsic control).

The Kaplan-Meir survival graphs in Figure 1 display the association of ER or PR expression with ovarian cancer specific survival. The presence and degree of expression of ER or PR were significantly associated with longer survival in EC (log rank *p* = 0.0005 and *p* = 0.0003, respectively). Other factors that were significantly associated with survival by univariate analysis was stage (*p* < 0.0001), residual tumor (*p* < 0.0001), and grade (*p* = 0.012).

The degree of ER or PR expression was significantly associated with ovarian cancer specific survival adjusted for age, center, stage, grade, and residual disease (Table 3). Although there was a marginally higher hazard ratio for diffuse staining compared to focal, this difference was not significant. Therefore, subsequent models included only presence or absence of staining. The presence of ER or PR were highly significantly associated with lower risk from death of EC. However, when stratified by stage, this effect was attenuated with only borderline significance for ER or both ER + PR for stage I/II. The multivariate models for stage III/IV disease were instable due to low numbers (*n* = 23) but univariate analysis shows that ER, PR, and ER + PR positive tumors were all associated with significantly longer survival (*p* < 0.0001 for all).

### 2.4. Clinical Utility of Hormone Receptor Expression Status

To assess whether hormone receptor expression status has added clinical utility for patient management, we performed analysis in subsets of patients defined by sub-stage. We considered a five-year OCSS of ≥95% as the cut-off where adjuvant chemotherapy could be withheld. Patients diagnosed at stage IA/IB exceeded this cut-off (97.8%). Here, only one patient out of 46 died of disease after 11 months and the pathology from this EC showed high-risk features of dedifferentiation. This tumor was ER/PR negative. The patient did not receive chemotherapy and disease recurred nine months after diagnosis as widely metastatic disease. Notably, 72% of patients with stage IA/IB disease did not receive adjuvant therapy including 27/36 grade 1, 4/5 grade 2, and 2/5 grade 3 all with favorable outcome. 

The five-year OCSS for all stage I ECs in our cohort was 94.1% (95% excluding the single dedifferentiated carcinoma). The largest subset consisted of stage IC and II with a five-year OCSS of 88.4% (90.0 for IC and 87.5% for II). In this subgroup, divergence of the survival curves was seen for ER positive tumors (five-year OCSS 89.0%, standard error 3.3%) compared to ER negative tumor (five-year OCSS 84.6%, standard error 12.0%, log rank *p* = 0.053), PR positive tumors (five-year OCSS 89.9%, standard error 3.2%) compared to PR negative tumor (five-year OCSS 80%, standard error 12.6%, log rank *p* = 0.11) and ER + PR positive tumors (five-year OCSS 89.5%, standard error 3.3%) compared to ER + PR negative tumor (five-year OCSS 80%, standard error 12.6%, log rank *p* = 0.11). However, hormone receptor status failed to identify an additional subset with five-year OCSS of ≥95%. Patients diagnosed at stage III had a five-year OCSS at 59.6% requiring adjuvant therapy.

### 2.5. Exploring Whether Hormone Receptor Status Might Be an Effect Modifier of Chemotherapy Response within Stage IC/II

**Figure 2:**
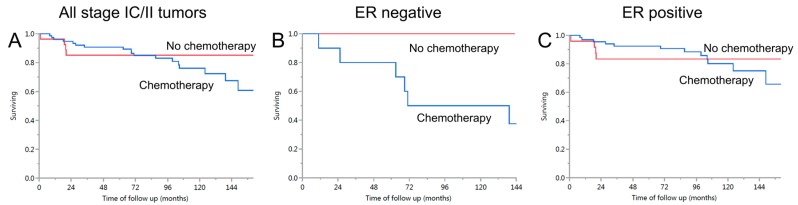
Patients with stage IC/II tumors: association between ER expression and benefit from adjuvant chemotherapy. (**A**) Survival of women diagnosed with ovarian endometrioid carcinoma stage IC/II who did (*n* = 76, blue line) or did not receive adjuvant chemotherapy (*n* = 27, red line), log rank *p* = 0.32; (**B**) Survival of women diagnosed with ovarian endometrioid carcinoma stage IC/II with ER negative tumors who did (*n* = 10, blue line) or did not receive adjuvant chemotherapy (*n* = 3, red line), log rank *p* = 0.19; (**C**) Survival of women diagnosed with ovarian endometrioid carcinoma stage IC/II with ER negative tumors who did (*n* = 66, blue line) or did not receive adjuvant chemotherapy (*n* = 24, red line), log rank *p* = 0.76.

We evaluated the association of receiving adjuvant chemotherapy with ovarian cancer specific survival stratified by hormone receptor expression status. There was no survival difference within all patients in stage IC/II nor when stratified by ER receptor expression status (Figure 2). In the small subgroup of ER negative tumor (*n* = 13, log rank *p* = 0.19), there was no statistical evidence that receiving chemotherapy was associated with longer survival. The results for PR and ER/PR combination were similar [15].

## 3. Discussion

Our results validate that hormone receptor positivity in EC is significantly associated with favorable five-year OCSS for women diagnosed with this disease [13]. Similar to the initial study, this is independent of other relevant risk factors such as stage and residual disease. This confirms the biological validity of an association of hormone receptor expression status with survival in EC using an independent set of samples “collected at a different point in time, at different institutions, from a different patient population, with samples processed in a different laboratory demonstrating the broad applicability” of this test, which adheres to the recommendation ‘1a’ from the Institute of Medicine on biomarker studies [16]. A strength of this study is that the analytical validity of the test has been established [17]. By using the same cut-off as previously, we were able to reproduce that the outcomes for cases with focal or diffuse ER expression overlap, PR expression shows a level dependent effect [13]. 

To be clinically useful, a prognostic marker should identify a subgroup with an excellent outcome so that additional therapy might be spared. We considered five-year ovarian cancer specific survival of 95% as such a threshold [3,18]. As previously shown, patients diagnosed with EC at stage IA/IB show a survival exceeding this threshold regardless of hormone receptor expression status [3,18]. This investigation adds to the literature the finding of a favorable long-term outcome among 46 stage IA/IB patients diagnosed with EC [3,18]. Only 26% of the 46 patients from our cohort received adjuvant chemotherapy confirming clinical practice that patients with EC at stage IA/IB do not require adjuvant therapy. The one patient who died of disease in this subgroup had a tumor showing high-risk histological features in the form of dedifferentiation, and those patients should be further studied. Recently, molecular alterations involved in dedifferentiation were discovered in the SNF/SWI complex (namely *SMARCA4* and *ARID1B*) [19]. With this information, a diagnosis of dedifferentiated carcinoma will likely become more reproducible in the near future, enabling better identification of those high risk patients.

We then investigated the next higher stages (IC/II). Although there were noticeable survival differences, ER or PR expression status did not identify an additional subgroup within stages IC/II with survival rates above the threshold, thereby limiting its clinical utility. For example, the five-year ovarian cancer specific survival for PR positive stage IC/II EC was 89.9% (standard error was 3.2), robustly lower than the 95% threshold. Rather, hormone receptor negativity singled out a small sub-group of cases with a more unfavorable outcome. This information might be used in multi-parameter prognostic models to identify patients that require more aggressive therapy. Future studies are warranted to determine the roles of ER and PR on differential regulation of proliferation/apoptosis and invasion in cultured ECs from ER/PR positive versus ER/PR negative ovarian endometrioid carcinomas or molecularly characterized endometrioid cell lines [20]. 

Interestingly, the five-year OCSS for all stage I EC in our cohort was 94% (95% excluding the single dedifferentiated carcinoma). Kumar et al. already extended the definition of low risk to include stage IC with surgical spill (FIGO 2014 IC1) [18]. In our cohort, there was no difference between the FIGO 2014 IC sub-stages, but there was a significant survival difference between stages I and II. Perhaps the line between low risk and intermediate risk group can be drawn between stages I and II, with the inclusion of stage IC into the low risk group. Reclassification of histotype might be a reason for the difference between the study by Kumar et al. and ours. We performed a multi-step histotype confirmation by full slide set review by a gynecological pathologist and application of diagnostic immunohistochemical markers. This avoided any influx of poor prognostic high-grade serous carcinomas, demonstrated by the low prevalence of combined WT1 expression and abnormal p53 expression [10] (only one case in the current cohort expressed this combination, but showed typical endometrioid morphology). 

The effects of treatment may confound prognostic marker studies in retrospective cohorts. In a stratified analysis for stage IC/II, we did not find a significant association of receiving adjuvant chemotherapy or not with ovarian cancer specific survival, a finding similar to the same histotype (high-grade endometrioid carcinoma) of the endometrium [21]. However, visual inspection of the Kaplan-Meier curves (Figure 2A) suggests that there is an approximate 10% difference from 24 to 72 months. We attempted to explore whether patients with hormone receptor negative EC would more likely benefit from chemotherapy. However, the case numbers in stage IC/II were small and a definitive conclusion cannot be made. This shows the limitation of subset analysis at the treatment threshold and supports the use of consortia data from several investigations to obtain the larger numbers of patients needed for studies in uncommon cancer types. Another limitation is that the clinical information was retrospectively abstracted from the medical chart. Some information was not reported in the medical chart, systematically resulting in a large proportion of missing values for certain variables. For example, surgical synoptic reporting including residual tumor status was only introduced a few years ago. 

While the prognostic information of hormone receptor expression has limited clinical utility, there is another implication which could be explored. We think that EC is an excellent candidate histotype of ovarian carcinoma to be considered for hormonal therapy based on the relative good outcome in stage I/II and a plausible biological rationale. Because of its negative feedback function, PR expression is viewed as a surrogate for a functionally active hormone axis [22,23]. Co-expression of hormone receptor expression is highly prevalent in EC, suggesting that the hormone axis is functionally active. Our finding of outcome differences further supports a biological role of hormone receptors in oncogenesis of most EC. In this study, we were not able to assess the predictive effect of hormone receptor expression status for hormonal therapy because it was not systematically administered. Hormonal therapy has been explored in ovarian carcinomas with mixed results. Meta-analyses reported an overall response rate of 13% with a complete response of 4% and stable disease of 38%. However, these trials neither stratified for histotype nor assessed the presence of the target (i.e., presence of hormone receptor expression) [24,25,26,27,28]. There is a suggestion that some subsets of ovarian carcinoma patients do benefit from hormonal therapy, and these might include ER/PR positive ECs [29,30,31,32,33]. Further studies of the efficacy of treating patients with EC with hormonal therapy are warranted under consideration of potential harms [34,35,36]. A potential limitation may be that hormone receptor-negative squamous morules could be hormonally inert impeding complete tumor eradication [37,38]. In the uterine counterpart, hormone receptor expression is also associated with a favorable prognosis, as shown by a meta-analysis of 98 studies [39]. In spite of absence of evidence from a prospective clinical trial, hormonal therapy is commonly administered to patients with recurrent low-grade endometrial carcinomas [27]. Notably, the survival association is not restricted to low-grade, but also seen in high-grade carcinomas of the endometrium [40]. 

EC shows a high frequency of hormonal receptor expression. Receptor negativity is associated with unfavorable outcome. Although the association is independent from known risk factors, this information does not add to current risk assessment. Ovarian cancers in general are usually not considered for hormonal therapy. The high prevalence of ER/PR co-expression and validated survival difference provides a rationale for further testing the efficacy of hormonal therapy in women diagnosed with hormone receptor positive ovarian endometrioid carcinoma, perhaps in scenarios such as maintenance therapy in stage IC/II disease or at recurrence. 

## 4. Materials and Methods

### 4.1. Study Cohort

Study cohort was obtained from the Alberta Ovarian Tumor Type (AOVT) study. The study was approved by the local Ethics board (REB15-1124). Case ascertainment was previously described in detail [6]. Briefly, participants were identified from the Alberta cancer registry, an accredited population-based registry, which records and maintains data on all new carcinoma patients and carcinoma deaths occurring within Alberta, Canada. AOVT was initiated to investigate rarer histotypes such as endometrioid, clear cell, and mucinous carcinomas. 

The study included nine ECs with focal clear cell features. These tumors were not classified as mixed carcinomas based on our recent findings that molecularly confirmed mixed ovarian carcinomas are exceptionally rare [41]. Immunohistochemistry supported their classification as endometrioid carcinoma with clear cell-like areas because of the absence of the clear cell marker Napsin A in seven of nine cases and the presence of ER expression in seven of nine cases. 

The pathology review of all ECs also assessed presence of squamous differentiation, grade (World Health Organization grading for endometrial endometrioid carcinoma), and presence of endometriosis in the surgical specimen. 

### 4.2. Clinical Information and Follow Up

Detailed chart abstraction and pathology review was performed as previously described [14]. The latest update was obtained on March 2015. The data included demographic characteristics of patient, date of surgery, presence or absent of residual disease, clinical stage, and CA125 levels. Type of treatment, regimen, and drugs were also recorded and time of disease recurrence and type of treatment given was also noted. With the exception of occasional Megestrol Acetate treatment in the palliative setting, hormonal therapy was not given to these patients. Information on follow up time was abstracted from patient records and calculated as the difference between the date of death and the date of diagnosis. The primary endpoint was ovarian cancer specific survival (OCSS), which was defined as death from ovarian cancer either presumed or with evidence of progression. Other causes of death unrelated to ovarian cancer (e.g., death from another cancer, non-cancer death) or treatment related were censored for the purpose of ovarian cancer related deaths.

### 4.3. Immunohistochemistry

Triplicate 0.6 mm tissue microarrays (TMA) were previously constructed [6], including normal tissue (tonsil, placenta, kidney, liver, and pancreas) as a control. Formalin fixed, paraffin embedded 4 µm TMA sections were deparaffinized and rehydrated. Heat-induced epitope retrieval was preformed on-board of the Leica BondRX platform at 100 °C using ethylenediaminetetraacetic acid (EDTA) buffer (pH 9.0, Leica, Buffalo Grove, IL, USA) for 20 min, followed by a 15-min incubation of ER (rabbit monoclonal anti-ER, clone SP1 recognizing ER alpha, 1:50 dilution, Thermo Fisher Scientific, Waltham, MA, USA) and PR (mouse monoclonal anti-PR, clone 16, recognizing the A form of human progesterone receptor by immunohistochemistry, prediluted, Leica Microsystems) at room temperature, post-primary reagent for 8 min, and Bond™ Polymer Refine Detection kit (Leica Microsystems, Inc., Buffalo Grove, IL, USA, DS9800) for 8 min. The reaction was visualized using 3,3-diaminobenzidine tetrahydrochloride for 10 min followed by hematoxylin as counterstain. Scoring was performed by a pathologist (MK or PR) on TMA glass slides at 20× magnification blinded to the clinical outcome using the same three tier scoring as in the study to be validated. This scoring system previously showed excellent inter-observer agreement of 91% and 92% for ER and PR, respectively [13]. Since squamous morules are commonly negative for ER and PR [36], interpretation was only performed in non-squamous tumor areas. Complete absence of nuclear stain was considered negative. Staining in 1% to 50% of tumor cell nuclei of any intensity was considered focal. Staining in more than 50% of tumor cell nuclei of any intensity was considered diffuse. Focal and diffuse staining was combined for analysis as staining present versus absent in negative cases. 

### 4.4. Statistical Analysis

Categorical variables were summarized as proportions while continuous variables were summarized as means or median with standard error depending on the data distribution. Survival probabilities were determined by the Kaplan-Meier method with display of survival curves, and comparison for groups was done by the log-rank test. Relative hazards between the groups were tested by the cox test. Cox proportional hazard regression models were used in multivariate analysis where ER and PR expression was used as the exposure. The data analysis was performed with statistical software STATA 13 (College Station, TX, USA) and JMP12 (SAS, Cary, NC, USA). The statistical tests were two-sided Chi-square for categorical data and two-sided student-*t* test for continuous data, and significant associations were considered when *p*-values were less than 0.05. The study adhered to the REMARK criteria [16].

## Figures and Tables

**Table 1:** Key patient characteristics and five-year ovarian cancer specific survival.

Parameter	*n* (%)	Five-Year Ovarian Cancer Specific Survival
**Centre**		
Calgary	93 (51.1%)	85.60%
Edmonton	89 (48.9%)	86.40%
**Stage**		
IA/IB	46 (25.3%)	97.80%
IC	40 (22.0%)	90.00%
II	64 (35.1%)	87.50%
III	21 (11.5%)	59.60%
IV	2 (1.1%)	0
Not documented	9 (5.0%)	76.20%
**Residual tumor**		
Absent	94 (51.6%)	89.00%
Present	15 (8.2%)	36.60%
Not documented	73 (40.1%)	91.80%
**Grade**		
1	125 (68.7%)	89.40%
2	23 (12.6%)	82.60%
3	34 (18.7%)	76.40%

**Table 2:** Association of progesterone receptor (PR) and estrogen receptor (ER) receptor expression with clinicopathological variables.

Variable	ER Absent	ER Present	*p*	PR Absent	PR Present	*p*
**Tumor Expression**, *n* (%)	23 (12.7)	158 (87.3)		24 (13.3)	157 (86.7)	
**Age at diagnosis**, Mean (SE)	52.8 (2.6)	54.1 (1.0)	0.63	54.6 (2.6)	53.8 (1.0)	0.79
**BMI ^1^**, Mean (SE)	25.8 (1.8)	29.9 (0.7)	0.040	25.8 (1.9)	29.8 (0.7)	0.051
**CA125**, Mean (SE)	428 (274)	549 (101)	0.68	748 (274)	505 (101)	0.40
**Endometriosis**, *n* (%)			0.42			0.54
Present	7 (30.4)	66 (41.8)		8 (33.3)	65 (41.4)	
Absent	16 (69.6)	90 (60.1)		16 (66.7)	90 (57.3)	
Unknown	0	2 (1.3%)		0	2 (1.3%)	
**Synchronous endometrial endometrioid carcinoma**, *n* (%)			0.45			0.38
Present	5 (21.4)	55 (34.8)		5 (20.8)	55 (35.0)	
Absent	11 (47.8)	61 (38.6)		11 (45.8)	61 (38.9)	
Unknown	7 (30.4)	42 (26.6)		8 (33.3)	41 (26.1)	
**Stage**, *n* (%)			0.57			0.70
IA/IB	4 (17.4)	41 (26.0)		5 (20.8)	40 (25.5)	
IC/II	13 (56.5)	91 (57.6)		15 (62.5)	89 (56.7)	
III	3 (13.0)	18 (11.4)		2 (8.3)	19 (12.1)	
IV	1 (4.4)	1 (0.6)		1 (4.2)	1 (0.6)	
Unknown	2 (8.7)	7 (4.4)		1 (4.2)	8 (5.1)	
**FIGO grade**, *n* (%)			0.0075			<0.0001
1	9 (39.1)	115 (72.3)		7 (29.2)	117 (74.5)	
2	6 (26.1)	17 (10.8)		9 (37.5)	14 (8.9)	
3	8 (34.5)	26 (16.5)		8 (33.3)	26 (16.6)	
**Squamous differentiation**, *n* (%)			0.0058			0.014
Absent	18 (78.3)	76 (48.4)		18 (75.0)	76 (48.7)	
Present	5 (21.7)	81 (51.6)		6 (25.0)	80 (51/3)	
**Treatment**, *n* (%)						
Residual tumor present	2 (8.7)	13 (8.2)	0.56	2 (8.3)	13 (8.3)	0.76
Radiation received	2 (8.7)	16 (10.1)	0.64	4 (16.7)	14 (8.9)	0.36
Chemotherapy received	16 (69.5)	99 (63.1)	0.65	17 (70.8)	98 (62.8)	0.59
**MMR ^2^**, *n* (%)			0.69			0.90
Deficient	2 (8.7)	23 (14.6)		3 (12.5)	22 (14.0)	
Proficient	21 (91.3)	134 (84.8)		21 (87.5)	134 (85.4)	
Missing	0	1 (0.6)		0	1 (0.6)	

^1^ BMI—body mass index; ^2^ MMR—mismatch repair.

**Table 3:** Cox-regression multivariate analysis for factors associated with survival.

Model	ER HR (95% CI)	PR HR (95% CI)	ER + PR HR (95% CI)
**Model 1 ^a^**	***n* = 172**	***n* = 172**	
Marker absent	1.0 (Reference)	1.0 (Reference)	Not performed
Marker focal	0.230 (0.0713–0.685)	0.256 (0.079–0.780)	
Marker diffuse	0.160 (0.07–0.403)	0.209 (0.089–0.517)	
*p*	0.0003	0.0018	
**Model 2 ^a^**	***n* = 172**	***n* = 172**	*n* = 160 *
Markers absent	1.0 (Reference)	1.0 (Reference)	1.0 (Reference)
Markers present	0.175 (0.076–0.425)	0.218 (0.096–0.527)	0.123 (0.066–0.428)
*p*	0.0002	0.0011	0.0001
**Model 3 ^b^ Stage I/II**	***n* = 149**	***n* = 149**	*n* = 138 *
Markers absent	1.0 (Reference)	1.0 (Reference)	1.0 (Reference)
Markers present	0.351 (0.137–0.976)	0.400 (0.151–1.176)	0.257 (0.079–0.946)
*p*	0.0452	0.0932	0.0417

^a^ adjusted for age, center, stage (missing stage excluded), residual disease (missing data as separate category), histological grade; ^b^ adjusted for age, center, residual disease (missing data as separate category), histological grade; * cases with either only ER or only PR expression were excluded.

## Abbreviations

EC	Endometrioid carcinoma
ER	Estrogen receptor
PR	Progesterone receptor
OCSS	Ovarian cancer specific survival
